# *Plasmodium falciparum* merozoite surface antigen-specific cytophilic IgG and control of malaria infection in a Beninese birth cohort

**DOI:** 10.1186/s12936-019-2831-x

**Published:** 2019-06-11

**Authors:** Rafiou Adamou, Célia Dechavanne, Ibrahim Sadissou, Tania d’Almeida, Aziz Bouraima, Paulin Sonon, Roukiyath Amoussa, Gilles Cottrell, Agnès Le Port, Michael Theisen, Edmond J. Remarque, Shirley Longacre, Kabirou Moutairou, Achille Massougbodji, Adrian J. F. Luty, Gregory Nuel, Florence Migot-Nabias, Ambaliou Sanni, André Garcia, Jacqueline Milet, David Courtin

**Affiliations:** 1 MERIT, IRD, Université de Paris, 75006 Paris, France; 2 Centre d’Etude et de Recherche sur le Paludisme Associé à la Grossesse et à l’Enfance, Faculté des Sciences de la Santé, Université d’Abomey-Calavi, Cotonou, Benin; 3 Laboratoire de Biochimie et de Biologie Moléculaire, Faculté des Sciences et Techniques, Université d’Abomey-Calavi, Abomey Calavi, Benin; 4 Laboratoire de Biologie et Physiologie Cellulaires, Faculté des Sciences et Techniques, Université d’Abomey-Calavi, Cotonou, Benin; 5 Division of Clinical Immunology, School of Medicine of Ribeirão Preto, University of São Paulo, São Paulo, Brazil; 6 Department for Congenital Disorders, Statens Serum Institut, Copenhagen, Denmark; 7 Centre for Medical Parasitology at Department of International Health, Immunology and Microbiology, University of Copenhagen, Copenhagen, Denmark; 8 Department of Infectious Diseases, Copenhagen University Hospital, Rigshospitalet, Copenhagen, Denmark; 9 Department of Parasitology, Biomedical Primate Research Centre, Rijswijk, The Netherlands; 10 Laboratoire de Vaccinologie-Parasitaire, Institut Pasteur, Paris, France; 11 Laboratoire de Probabilités et Modèles aléatoires (LPMA), UMR CNRS 7599, UPMC, Paris, France

**Keywords:** *Plasmodium falciparum*, Malaria, Cytophilic IgG, Merozoite vaccine candidate antigens

## Abstract

**Background:**

Substantial evidence indicates that cytophilic IgG responses to *Plasmodium* *falciparum* merozoite antigens play a role in protection from malaria. The specific targets mediating immunity remain unclear. Evaluating antibody responses in infants naturally-exposed to malaria will allow to better understand the establishment of anti-malarial immunity and to contribute to a vaccine development by identifying the most appropriate merozoite candidate antigens.

**Methods:**

The study was based on parasitological and clinical active follow-up of infants from birth to 18 months of age conducted in the Tori Bossito area of southern Benin. For 399 infants, plasma levels of cytophilic IgG antibodies with specificity for five asexual stage malaria vaccine candidate antigens were determined by ELISA in infants’ peripheral blood at 6, 9, 12 and 15 months of age. Multivariate mixed logistic model was used to investigate the association between antibody levels and anti-malarial protection in the trimester following the IgG quantification. Moreover, the concentrations of merozoite antigen-specific IgG were compared between a group of infants apparently able to control asymptomatic malaria infection (CAIG) and a group of infants with no control of malaria infection (Control group (NCIG)). Protective effect of antibodies was also assessed after 15 months of malaria exposure with a Cox regression model adjusted on environmental risk.

**Results:**

Cytophilic IgG responses to AMA1, MSP1, MSP2-3D7, MSP2-FC27, MSP3 and GLURP R2 were associated with increasing malarial infection risk in univariate analysis. The multivariate mixed model showed that IgG1 and IgG3 to AMA1 were associated with an increased risk of malarial infection. However infants from CAIG (n = 53) had significantly higher AMA1-, MSP2-FC27-, MSP3-specific IgG1 and AMA1-, MSP1-, MSP2-FC27-, MSP3 and GLURP-R2-specific IgG3 than those from NCIG (n = 183). The latter IgG responses were not associated with protection against clinical malaria in the whole cohort when protective effect is assessed after 15 months of malaria exposition.

**Conclusion:**

In this cohort, merozoite antigen-specific cytophilic IgG levels represent a marker of malaria exposure in infants from 6 to 18 months of age. However, infants with resolution of asymptomatic infection (CAIG) seem to have acquired naturally immunity against *P. falciparum*. This observation is encouraging in the context of the development of multitarget *P. falciparum* vaccines.

## Background

*Plasmodium falciparum* malaria remains a significant cause of infant mortality and morbidity in many parts of the world especially in sub-Saharan Africa. In 2017, there were an estimated 219 million cases of malaria and 435,000 deaths [1]. In endemic countries, children under 5 years are particularly vulnerable to malaria. During repeated exposure to infected *Anopheles* bites, infants develop immune responses that reduce clinical symptoms and life-threatening complications. Antibodies are known to be key components of naturally-acquired anti-malarial [2] most notably in studies involving passive transfer of antibodies from immune adults to malaria-infected children that resulted in reductions of both parasitaemia and clinical symptoms [2, 3]. Among IgG subclasses, cytophilic IgG1 and IgG3 are thought to play a key role in anti-malarial protection [4, 5]. However, it is unclear which merozoite antigens may be the most important targets of naturally-acquired immunity [6].

An efficacious blood stage vaccine against malaria that remains potent in different transmission settings would greatly contribute to reducing the disease burden among endemic populations. RTS,S/AS01 (Mosquirix^®^) is the most advanced malaria vaccine, targeting pre-erythrocytic stages, but it is suboptimal both in terms of efficacy and duration [7, 8]. Developing new and more potent malaria vaccines is, therefore, a priority and sero-epidemiological studies are one of the most valuable tools that can be used to identify naturally-acquired protective anti-malarial antibody (Ab) responses.

To date, a number of *P.* *falciparum* merozoite antigens have been assessed in human vaccine trials including the most studied antigens: merozoite surface protein (MSP) 1, 2, 3, apical membrane antigen 1 (AMA1) and glutamate rich protein (GLURP) [6]. Numerous antibody responses targeting *P.* *falciparum* merozoite surface proteins have been associated with reduced risk of malaria and high parasitaemia in naturally-exposed children or adults [9–19]. However a number of immuno-epidemiological studies conducted in endemic communities have provided conflicting data, with antibody responses to the same antigens appearing to be protective against malaria in some studies but associated with a higher risk of malaria in others [13, 17, 20–32]. The reasons for discordant observations could be numerous and most likely include specific differences in malaria transmission patterns (unstable, stable), study design (active or passive follow-up), population under study (infants, children, adults, host genetic diversity) and technical aspects (differences in operating procedure and methods of data analysis). The study of antibody responses to merozoite candidate vaccine antigens in infant cohorts are of interest as young children are the main victims of *P.* *falciparum* and they represent the main target in any malaria vaccination strategy. Previous work conducted in the Tori-Bossito cohort showed that factors such as age, past and current malaria infections, malaria antibody levels at birth, as well as exposure to *Anopheles* bites were significantly associated with the natural acquisition of anti-malarial IgG1 and IgG3 responses in 6- to 18-month old infants [33]. In the same cohort, the present study aimed to investigate the role of the cytophilic antibody responses acquired against specific *P.* *falciparum* candidate vaccine antigens in protection of infants against *P.* *falciparum* infection. IgG1 and IgG3 responses to five leading *P.* *falciparum* merozoite-stage vaccine candidate antigens (AMA1, MSP1, MSP2, MSP3, and GLURP) were quantified to investigate associations between these antibody responses and anti-malarial protection from 6 to 18 months of age.

## Methods

### Study design

The data here were collected from a longitudinal malaria cohort study conducted in 9 villages of the district of Tori Bossito located in the Southern part of Benin described in detail elsewhere [34]. Briefly, 656 newborns were enrolled between June 2007 and January 2010 and both clinical and parasitological follow-ups were performed from birth to 18 months of age. Mothers were invited to bring their infants to the health centre at any time in case of suspicious fever or clinical signs, whether related to malaria or not. In case of fever, a questionnaire and both a rapid diagnostic test (RDT) and a thick blood smear (TBS) were performed. Symptomatic malaria infection (fever or history of fever in the preceding 48 h and positive TBS and/or RDT) was treated with the artemether–lumefantrine combination, as recommended by the National Malaria Control Programme. The same procedure was applied for each child who was visited every week by a nurse of the programme during the entire follow-up. Moreover a systematic TBS was performed monthly to detect malaria infection.

During systematic visits *P. falciparum* infection could be symptomatic or asymptomatic (absence of fever or history of fever in the preceding 48 h, presence of *P. falciparum* and absence of symptomatic infections in the following 3 days). All blood smears were stained with 10% Giemsa and were read by two independent technicians. A third reading was performed when discordant cases occurred.

For each woman at delivery, a questionnaire collecting information on maternal age, parity, use of Intermittent Preventive Treatment during pregnancy (IPTp) and bed net possession was administered. In addition, thick and thin placental smears were made to detect placental malaria. An entomological, nutritional and biological follow-up was also performed as described in detail elsewhere [35]. During the study, the annual *P.* *falciparum* entomological inoculation rate was calculated in all villages, giving an average of 15.5 infected *Anopheles* bites/person/year [35], with large spatial and temporal variations between the villages. A predictive regression model using entomological and environmental data was developed to predict the spatiotemporal variability of malaria transmission within the nine villages [35]. Hence, a time-dependent environmental risk of exposure (ERE) was attributed to each child included in the cohort. A questionnaire concerning nutrition was administered by supervisors to collect information about breastfeeding and to assess the quality of feeding practices through a qualitative dietary 24 h recall. An individual dietary diversity score was created according to Food and Agriculture Organization recommendations [34, 36]. Also, a time-dependent quantitative variable accounting for the past 3-month feeding practices was created [34].

Infant blood samples were collected in EDTA vials every 3 months during the follow-up and plasmas were conserved at − 80 °C for antibody quantification. In the present study, the main objective was to assess the protective role of infant antibody responses to malaria infection every 3 months following the blood draw. Antibody measurements at 0 and 3 months of age were excluded because of the content in those samples of antibodies from mixed maternal and infant origins. Therefore, only antibody measurements at 6, 9, 12 and 15 months were processed.

### Recombinant antigens

The plasmodial antigens used in this study included a recombinant AMA1 (amino-acids 25–545, FVO strain), which was expressed in *Pichia pastoris* and was produced by the Biomedical Primate Research Centre (Rijswijk, The Netherlands) [37]. MSP1_19_ (Uganda-Palo-Alto strain) was expressed in a Baculovirus/insect cell system [38]. It is composed of 2 combined long peptides (amino acids 1–43 and 1615–1723) and was produced at Pasteur Institute of Paris. MSP2 (3D7 and FC27, whole proteins without the secretion signal and the GPI anchorage) were the gift of collaborators from La Trobe University (Melbourne, Australia) [39, 40]. GLURP-R0 (amino acids 25–514, F32 strain), GLURP-R2 (amino acids 706–1178, F32 strain) and MSP3 (amino acids 212–380, F32 strain) were produced by the Infection-Immunity Department of the Statens Serum Institute of Copenhagen (Denmark) and were expressed in *Escherichia coli*.

### Antibody measurements

The standard operating procedures developed by the African Malaria Network Trust was used to assess cytophilic IgG1 and IgG3 concentrations by enzyme linked immunosorbent assay (ELISA) to a panel of recombinant proteins, as described previously [13]. Briefly, recombinant proteins (0.1 µg/well) diluted in phosphate buffered saline (PBS) were coated on MaxiSorp Nunc plates (Thermo Fisher Scientific, Denmark) and blocked with 3% powdered-milk 0.1% PBS-Tween 20. Plasma samples were diluted 1:50 for all recombinant proteins. Peroxidase conjugated anti-human IgG1 (NL16 clone) diluted 1:2000 and anti-human IgG3 (ZG4 clone) diluted 1:5000 (Skybio, France) were used for revealing the reaction with 3,3′,5,5′-tetramethylbenzidine (TMB) as substrate. Standard curves were established using human IgG1 and IgG3 purified proteins (Binding Site, France) to determine the concentration of specific antibodies. Each point was tested in duplicate.

### Management of ELISA data

R script based on ADAMSEL software (Auditable Data Analysis and Management System for ELISA) [41] was used to transform optical density (OD) values into antibody concentrations. Discordant duplicates (with a variation coefficient > 15%) were retreated. OD that were below detection threshold or over saturation were referred to as “Low” and “High” concentration values (µg/mL) respectively. A stochastic expectation maximization algorithm [42] already applied to ELISA analyses [43] was used to impute data in these particular cases taking into account the lowest, the highest and the standard concentration values of the ELISA plates in which the Low/High OD values were recorded.

### Analysis strategy

In the first approach, a logistic mixed model was performed to study the association between the antibody levels quantified at 6, 9, 12 and 15 month of age for 399 infants and the risk of malarial infection from 6 to 18 month of age. Each quarterly antibody measurement was associated with the presence or absence of at least one malaria infection during the following 3 months in the logistic mixed model. To focus specifically on antibody-mediated effects, the cytophilic IgG levels were first adjusted using a linear regression on the nuisance variables (prematurity, parity, use of IPTp, bed net possession, health centre and maternal age). The residuals of these adjustments were then used in the models. Moreover, all analyses were adjusted on maternal and newborn’s covariates known to potentially influence malarial infection: placental malaria, parity, prematurity, haemoglobin levels at delivery and birth, administration of IPTp, birth weight, ethnicity, age, environmental risk of exposure to malaria, gender, nutrition status, sickle cell trait and bed net use. Multivariate analysis was performed including the variables with *p* ≤ 0.10 in the univariate analysis.

In the second approach, a linear mixed model was used to assess the difference of the levels of cytophilic IgG between a group of infants apparently able to control asymptomatic malaria infections (CAIG (n = 53)) and a control group (NCIG (n = 183)) of infants infected by *P.* *falciparum* without resolution of asymptomatic malaria infection. The control of asymptomatic infection was defined here as the disappearance of asymptomatic infections over time based on TBS examination. Infants from CAIG seem able to control not only the disease symptom (asymptomatic infection) but also the parasitaemia. NCIG was composed of infants at least exposed to two malaria infections during the follow-up. No difference in term of environmental risk of exposure to malaria (ERE variable) was observed between both groups. It is important to notice that the linear mixed model took into account IgG cytophilic levels at 6, 9, 12 and 15 months, respectively.

A linear mixed model was used to test the association between the antibody quantification at 15 months and the parasite density in the following quarter. The mean of the parasite density between 15 and 18 months (all infections combined) was calculated and antibody levels were divided into 4 categories according to the quartiles.

Finally, a Cox proportional hazards regression model was used to test the protective effect of IgG responses previously associated with the phenotype of asymptomatic infection clearance (CAIG) at 15 months of age in the whole sample (CAIG + NCIG). Hazard ratios (HR) of clinical malaria were calculated on the 15–18 months period adjusted by ERE. To avoid a bias due to anti-malarial therapy, it has been considered a 14 days period after treatment where the infants were not considered at risk. Thus for infants who had a symptomatic malaria infection at blood sampling (n = 14) or within the 2 weeks before blood sampling, the starting point of survival analysis was shift to 14 days after treatment (n = 28). Those with an asymptomatic infection at sampling (n = 19) were excluded from analysis.

All statistical analyses except Cox model were performed using Stata, version 13.0 (StatCorp LP, TX, USA). Cox model were performed using the R package “survival” [44]. Statistical significance was set at *p *< 0.05.

### Ethics

The study protocol was approved by the Ethics Committee of the University of Abomey-Calavi (Faculté des Sciences de la Santé) in Benin and the Consultative Committee of Ethics of the Institut de Recherche pour le Développement. All women signed an informed consent before enrollment (which also included their children). All the methods were carried out in accordance with the approved guidelines.

## Results

### Characteristics of study participants

Newborns for whom plasma samples and clinical and parasitological data were available were included in the analyses (n = 399). Eighty-two per cent of the mothers declared having received IPTp at least once (sulfadoxine–pyrimethamine is the treatment recommended by the Beninese National Malaria Control Programme). Fourteen per cent of them were primigravidae and 10% had an infected placenta at delivery. Eleven per cent of the newborns had a low birth weight (< 2500 g) and 12% were born prematurely. The sex ratio was 0.98 and infants followed in the study belonged to different ethnic groups: Tori and Fon were the principal ethnicities represented with minorities of Aïzo and Yoruba. Sickle-cell trait (HbAS) carriers accounted for 22% of the study population. Details of the study participants are presented in Table 1.

**Table 1:** Characteristics of the participants

Individuals (n = 399)	Variables*	Characteristics (%; n)
Mothers	Intermittent preventive treatment (n = 399)	Yes: 82% (327) No: 18% (72)
	Gravidity status (n = 399)	Primigravidity: 15% (58) Multigravidity: 85% (341)
	Placental malaria (n = 383)	Yes: 10% (37) No: 90% (346)
Infants	Low birth weight (< 2500 g) (n = 399)	Yes: 12% (46) No: 88% (353)
	Prematurity (n = 399)	Yes: 11% (45) No: 89% (354)
	Gender (n = 399)	Female: 50% (201) Male: 50% (198)
	Ethnicity (n = 390)	Tori: 74% (289) Fon: 10% (39) Others: 16% (62)
	Bed net possession (n = 399)	Yes: 65% (258) No: 35% (141)
	HbAS (n = 381)	Yes: 23% (87) No: 77% (294)

*Data on different variables are not available for all individuals

A total of 834 uncomplicated malaria attacks (UMA) (from 1 to 11 UMA/infant) and of 168 asymptomatic infections (from 1 to 8 asymptomatic infections/infant) were recorded during the follow-up. Based on the definition (cf. “Methods”), 53 infants were included in CAIG and 183 infants in NCIG (Table 2).

**Table 2:** Clinical and parasitological characteristics for infants in CAIG and NCIG groups

	Malaria infection groups^a^	
	Asymptomatic infection clearance without anti-malarial treatment group (CAIG) N = 53	Control group (NCIG) N = 183
Infections during the follow-up asymptomatic infections, no (%)		
No infection	0	121 (66)
1	24 (45)	48 (26)
2–3	29 (55)	13 (7)
> 3	0	1 (0)
Symptomatic infections, no (%)		
No infection	6 (11)	0 (0)
1	17 (32)	26 (14)
2–3	18 (34)	102 (56)
> 3	12 (23)	55 (30)
Parasite density^b^ in asymptomatic infections, median (IQR)	38.6 (6.2–143.9)	60.2 (6.4–168.8)
Parasite density in symptomatic infections, median (IQR)	255.7 (8.0–806.8)	180.3 (10.1–698.8)
Time until malaria attacks (days)^c^, median (25th–75th)	34 (26–47)	13.5 (6–31)
Environmental risk of exposure to malaria^d^ during the follow-up, median (25th–75th)	3.06 (2.15–4.60)	3.89 (2.54–5.70)

^a^CAIG was composed by 53 infants with spontaneous clearance of at least one asymptomatic infection. Among these 53 infants, six never had febrile malaria during the follow-up while 47 also developed at least one malaria attack. Among the 168 asymptomatic infections, 88 and 80 respectively occurred in CAIG and NCIG during the whole follow-up. 64/88 were followed by a negative of thick blood smears in the time, 17/88 were followed by a malaria attacks and 7/88 occurred at the end of the follow-up for which it was not possible to determine the outcome. Concerning NCIG, 62/80 were followed by a malaria attacks and 18/80 occurred at the end of the follow-up for which it was not possible to determine the outcome

^b^Parasite density was expressed in number of *Pf*-infected red blood cells for 100 leucocytes

^c^The time expressed in days between the detection of asymptomatic infection and the occurrence of febrile malaria was higher in CAIG than NCIG. All infants (n = 30) with only one malaria infection were considered as not sufficiently exposed to belong to both groups and excluded

^d^Environmental risk of exposure to malaria was estimated for each infant once a month. No significant difference of exposure to malaria was observed between infants from CAIG and NCIG (p = 0.11; Mann–Whitney test)

### Anti-malarial IgG levels according to the risk of malaria infection

In this analysis, the association between specific IgG levels and protection to malaria was investigated. Univariate analyses showed that both IgG1 and IgG3 levels quantified at 6, 9, 12 and 15 months of age were significantly associated with increasing risk of malarial infection within the 3 months after blood draw (*p *< 0.05) with the exception of antibody responses to GLURP R0 (Table 3). Placental infection, ethnic group, environmental risk of exposure to malaria, nutritional status and age were also associated with malarial infection (*p *< 0.10). None of the other covariables showed an association with the risk of infection (Table 3).

**Table 3:** Factors associated with malarial infection from 6 to 18 months of age (univariate analysis with logistic mixed model)

Variables	OR	*p*	CI 95%
Placental malaria	1.91	*0.023*	[1.09,3.34]
Multigravida	0.85	0.521	[0.52,1.38]
Prematurity	0.86	0.611	[0.50,1.50]
Maternal Hb level at delivery	0.94	0.347	[0.83,1.06]
IPTp intake	0.84	0.443	[0.53,1.31]
Ethnic groups*			
Fon	1.08	0.789	[0.60,1.95]
Others	0.53	*0.016*	[0.32,0.89]
Low birth weight	0.87	0.637	[0.51,1.50]
Environmental risk	1.21	*<* *0.001*	[1.16,1.27]
Female	1.22	0.251	[0.86,1.72]
Infant Hb level at birth	0.99	0.883	[0.91,1.08]
Nutritional status	1.59	*0.056*	[0.98,2.58]
HbAS	0.94	0.802	[0.62,1.44]
Bed net possession	0.76	0.138	[0.53,1.09]
Age (6 month at reference)			
9	1.90	*0.001*	[1.29,2.80]
12	4.58	*<* *0.001*	[3.11,6.75]
15	6.04	*<* *0.001*	[4.08,8.94]
Antibody			
IgG1 to AMA1	1.08	*0.004*	[1.02,1.14]
IgG1 to MSP1	1.08	*0.001*	[1.03,1.14]
IgG1 to MSP2 3D7	1.09	*0.001*	[1.03,1.15]
IgG1 to MSP2 FC27	1.06	*0.032*	[1.00,1.12]
IgG1 to MSP3	1.13	*0.002*	[1.05,1.22]
IgG1 to GLURP R0	1.07	0.052	[0.99,1.14]
IgG1 to GLURP R2	1.07	*0.028*	[1.00,1.14]
IgG3 to AMA1	1.09	*0.002*	[1.04,1.17]
IgG3 to MSP1	1.06	*0.020*	[1.01,1.13]
IgG3 to MSP2 3D7	1.05	*0.026*	[1.00,1.11]
IgG3 to MSP2 FC27	1.05	*0.038*	[1.00,1.11]
IgG3 to MSP3	1.10	*0.007*	[1.02,1.18]
IgG3 to GLURP R0	1.01	0.667	[0.94,1.10]
IgG3 to GLURP R2	1.07	*0.018*	[1.01,1.15]

Significant *p* values (*p *≤ 0.10) are mentioned in italics

OR: odds ratio; OR > 1: variables associated with higher risk of malarial infection from 6 to 18 months of age, OR < 1: variables associated with lower risk of malarial infection from 6 to 18 months of age, *p*: p-value, CI: confidence interval

*Tori, Fon and other less represented ethnic groups (Aïzo, Yoruba) were present in the study area: Tori were used as the reference group in the analysis. In the present analysis, the level of IgG1 and IgG3 quantified at each plasma collection (6, 9, 12 and 15 month of age) was correlated with malarial infection in the following trimester. Similar results were obtained when analysis was performed using data for symptomatic infections only

Covariables associated with malarial infection (*p *< 0.10) in univariate analysis were included in the multivariate analysis (Table 4). Given that a strong correlation exists between IgG1 and IgG3 responses, a top-down multivariate model was applied separately for both isotypes. Environmental risk and age variables remained positively associated with the risk of malarial infection from 6 to 18 months of age. IgG1 and IgG3 to AMA1 were associated with an increased risk of infection with *P.* *falciparum* within the 3 months after blood draw (*OR *= 1.07; *p *= 0.039 and *OR *= 1.09; *p *= 0.021, respectively) (Table 4).

**Table 4:** Association between antibody levels and the risk of malaria infection from 6 to 18 months of age (multivariate analysis with logistic mixed model)

Variables	OR	*p*	CI (95%)
IgG1 to AMA1	1.07	*0.039*	[1.00,1.15]
Placental malaria	2.03	*0.043*	[1.02,4.06]
Environmental risk	1.22	*<* *0.001*	[1.16,1.29]
Nutritional status	1.36	0.297	[0.76,2.46]
Age (months)^a^			
9	1.97	*0.004*	[1.25,3.11]
12	4.91	*<* *0.001*	[3.08,7.81]
15	6.90	*<* *0.001*	[4.31,11.05]
Ethnic group^b^			
Fon	0.96	0.926	[0.46,2.01]
Others	0.51	*0.037*	[0.27,0.96]
IgG3 to AMA1	1.09	*0.021*	[1.01,1.18]
Placental malaria	2.03	*0.045*	[1.01,4.07]
Environmental risk	1.22	*<* *0.001*	[1.16,1.29]
Nutrition status	1.48	0.193	[0.81,2.69]
Age (months)			
9	1.89	*0.006*	[1.20,2.99]
12	5.10	*<* *0.001*	[3.20,8.12]
15	7.18	*<* *0.001*	[4.49,11.50]
Ethnic group			
Fon	0.90	0.790	[0.43,1.88]
Others	0.49	*0.031*	[0.26,0.93]

Significant *p* values (*p *< *0.05*) are mentioned in italics

OR: odds ratio; OR > 1: variables associated with higher risk of malarial infection from 6 to 18 months of age; OR < 1: variables associated with lower risk of malarial infection from 6 to 18 months of age; *p*: p-value; CI: confidence interval

^a^6, 9, 12 and 15 months quantifications were considered in analyses and 6 month quantification was used as the age of reference

^b^Ethnic groups were Tori, Fon and other less represented ethnic groups (Aïzo, Yoruba, …). Tori was used as the reference group in the analyses

### Anti-malarial IgG levels according to malaria symptomatology and parasitaemia

**Fig. 1:**
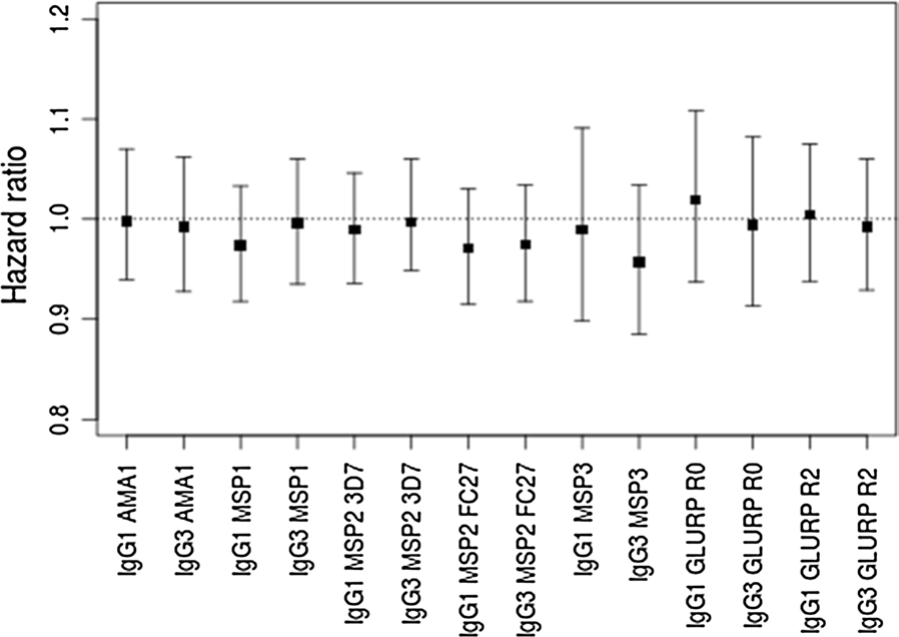
Hazard ratios and the 95% confidence interval of clinical malaria for IgG1 and IgG3 levels for children at 15 months. A Cox proportional hazards regression model was used to test the protective effect of IgG responses previously associated with the phenotype of asymptomatic infection clearance at 15 months of age in the whole sample (CAIG + NCIG). Hazards ratios obtained from Cox-regression model adjusted by environmental risk of exposure to malaria, considering exposed children with no asymptomatic infection at the time of Ab measurement. The number of infants included in Cox-regression model was 207 (13 infants had no Ab measurement at 15 months of age and 19 infants were excluded due to presence of asymptomatic infection at the time of Ab measurement). 66, 81, 50, 9 and 1 infants had, respectively, 0, 1, 2, 3 and 4 malaria attacks between 15 and 18 months of follow-up

Individuals belonging to the CAIG group had higher antibody levels compared to those from the NCIG group. Thus, their levels of AMA1-, MSP2-FC27-, MSP3-specific IgG1 and AMA1, MSP1, MSP2-FC27, MSP3, GLURP-R2-specific IgG3 were significantly higher (*p *≤ 0.05), whilst their levels of IgG3 with specificity for MSP2-3D7 displayed trends towards higher levels (*p *= 0.067) (Table 5). Then, the assessment of the protective effect of Ab responses previously associated to CAIG in the whole cohort at 15 month of age have been done considering that protection could emerge only at the end of the follow-up. Cox proportional hazards regression model showed no association between Ab responses previously associated with asymptomatic infection clearance without anti-malarial treatment on the 15–18 months period (Fig. 1). Antibody titers quantified at 15 months of age were not associated with a modulation of parasite density in the following 3 months.

**Table 5:** Comparison of IgG levels in NCIG and CAIG

	Comparison of IgG level in NCIG and CAIG			Age			ERE^a^		
	Coef.	*p*	CI 95%	Coef.	*p*	CI 95%	Coef.	*p*	CI 95%
IgG1 to AMA1	0.82	*0.005*	[0.25,1.40]	0.10	< *0.001*	[0.06,0.15]	0.08	< *0.001*	[0.04,0.13]
IgG1 to MSP1	0.47	0.129	[− 0.13,1.07]	0.11	< *0.001*	[0.06,0.16]	0.15	< *0.001*	[0.10,0.19]
IgG1 to MSP2 3D7	0.46	0.112	[− 0.11,1.04]	0.10	< *0.001*	[0.05,0.15]	0.14	< *0.001*	[0.09,0.18]
IgG1 to MSP2 FC27	0.59	*0.042*	[0.02,1.16]	0.09	< *0.001*	[0.04,0.14]	0.12	< *0.001*	[0.07,0.16]
IgG1 to MSP3	0.50	*0.013*	[0.10,0.91]	0.04	*0.004*	[0.01,0.07]	0.04	*0.011*	[0.009,0.07]
IgG1 to GLURP R0	0.20	0.361	[− 0.23,0.64]	0.02	0.177	[− 0.23,0.64]	0.024	0.154	[− 0.09,0.06]
IgG1 to GLURP R2	0.36	0.163	[− 0.14,0.86]	0.04	*0.026*	[0.005,0.08]	0.09	< *0.001*	[0.05,0.13]
IgG3 to AMA1	0.78	*0.003*	[0.27,1.29]	0.09	< *0.001*	[0.06,0.14]	0.11	< *0.001*	[0.07,0.15]
IgG3 to MSP1	0.81	*0.003*	[0.27,1.36]	0.07	*0.003*	[0.02,0.12]	0.16	< *0.001*	[0.11,0.20]
IgG3 to MSP2 3D7	0.57	0.067	[− 0.04,1.18]	0.15	< *0.001*	[0.10,0.19]	0.11	< *0.001*	[0.07,0.16]
IgG3 to MSP2 FC27	0.66	*0.023*	[0.09,1.24]	0.14	< *0.001*	[0.09,0.19]	0.13	< *0.001*	[0.08,0.17]
IgG3 to MSP3	0.64	*0.002*	[0.23,1.05]	0.05	*0.002*	[0.019,0.08]	0.07	< *0.001*	[0.04,0.10]
IgG3 to GLURP R0	0.16	0.426	[− 0.23,0.55]	0.03	*0.034*	[0.002,0.06]	0.06	< *0.001*	[0.03,0.09]
IgG3 to GLURP R2	0.53	*0.021*	[0.08,0.98]	0.06	*0.003*	[0.02,0.09]	0.10	< *0.001*	[0.06,0.14]

Multivariate analyses (linear mixed models) were performed to compare the difference of Ab mean level between both groups taking into account the Ab measurements at 6, 12, 15 and 18 months of age

Models were adjusted by age and environmental risk. Ab level from CAIG was considered as the reference in the model

Significant *p* values (*p *< 0.05) are mentioned in italics

Coef.: coefficient, Coef. > 0: indicate a higher level of Ab from 6 to 18 months of age in CAIG group compared to NCIG group, CI: confidence interval

^a^ERE represent environmental risk of exposure attributed quarterly for each infant included in the cohort

## Discussion

After birth, infants exposed to repeat malaria infections gradually acquire anti-malarial antibodies while their maternal antibodies wane in time. Numerous antigens on the merozoite surface have been identified as important targets of naturally-acquired immunity, and the need to develop an effective blood-stage vaccine against *P.* *falciparum* remains a research priority. In the present birth cohort study, unique in its multidisciplinary approach [34], its sample size and its closed follow-up, antibody responses to *P.* *falciparum* merozoite vaccine candidates were studied. The main objective of the present work was to evaluate whether cytophilic IgG acquired during the first 6- to 18-month period of life was sufficient to protect infants against *P.* *falciparum* malaria. The key findings showed that (i) the level of cytophilic antibodies directed to AMA1 were associated with an increasing risk of malarial infection during the complete cohort follow-up and (ii) a group of infants with resolution of *P.* *falciparum* asymptomatic infections (CAIG) had higher cytophilic IgG levels to AMA1, MSP1, MSP2-FC27, GLURP-R2 and MSP3.

First result suggests that IgG1 and IgG3 responses to AMA1—quantified every 3 months in all infants of the cohort—represent more a biomarker of exposure than a biomarker of protection from malaria. Most of the results observed in other infant cohorts have revealed similar associations between the antibody level and an increased risk of malaria. In a cohort study conducted by Kusi in Ghana on infants between 1 and 5 years old, authors found that anti-AMA1 antibodies were associated with a history of infection [45]. Stanisic et al. investigated the role of antibodies to a panel of vaccine candidate antigens including AMA1 in cohorts of 1–4 and 5–14 year-old children in Papua New Guinea and found that children with higher antibody levels had an increased risk of malaria compared to those with low or no detectable anti-malarial antibodies [31]. Another study conducted in Kenya showed that presence of detectable anti-malarial antibodies at 12 months of age was predictive of a higher risk of malaria in the subsequent year of life [46]. Kangoye et al. found that antibodies to AMA1 and MSP1_19_ were significantly associated with an increasing malaria risk in two cohorts, each constituted by 40 children aged from 4 to 6 weeks in Burkina Faso and Senegal [18]. Other studies that focused on acquired anti-malarial antibodies failed to show the protective role of these antibody responses against malaria attacks [13, 14]. The authors hypothesized that antibody responses acquired by infants and young children through malaria exposure failed to reach a critical “protective” threshold until 4 years or older.

In order to better understand the acquisition of IgG to vaccine candidates leading to protection, a group of infants that experienced asymptomatic infections followed by a disappearance of these asymptomatic infections during the follow-up (CAIG) was defined. CAIG definition was based on microscopic examination of TBS although the sensitivity of this method is questionable. However, submicroscopic infections, even detectable by amplification of *P. falciparum* DNA could not be sufficient to prove the viability of the parasite as demonstrated by different groups [47]. It could also not be exclude the possibility that some of these infants have taken a malaria treatment however it is unlikely for different reasons. First, these infections were asymptomatic. Secondly, the mothers were invited to bring their infants to the health centre for free care at any time in case of fever or clinical signs and the information concerning malaria detection and its treatment were recorded. Finally, each child was visited every week by health workers at home during the entire follow-up to check his health status and collect information related to malaria. Based on this definition (i.e. including its limits), a second result showed that higher levels of cytophilic IgG to AMA1, MSP1, MSP2-FC27, GLURP-R2 and MSP3 were observed in CAIG than in the control group composed of infants similarly exposed to malaria infection. These findings suggest that a non-negligible proportion of infants (13%) seem to acquire a form of immunity associated with a control of asymptomatic malaria infection. These results also suggest that a certain quantity of specific IgG is needed to be able to control the infection. This result is in line with studies, which reported associations between anti-malarial protection and levels of antibodies directed to blood stage vaccine candidate antigens in young children. Murungi et al. found that anti-AMA1 antibodies were associated with a significant reduction in the odds of developing severe malaria in a unique prospective matched case–control study including Kenyan children aged 0 to 2 years [19]. Another study conducted by Nebie et al. in children aged more than 6 months old in Burkina Faso showed that IgG1 responses to AMA1 were associated with protection against malarial infection [12]. AMA1 is known for its high immunogenicity and this characteristic may explain why IgG to AMA1 is often found associated with the malaria status during infancy.

Antigenic variations are often reported and often alter the vaccine efficacy. Therefore, potential differences between the strains used to produce the recombinant proteins and the *Plasmodium* strains circulating in this Beninese study area could lead to an underestimation of antibody responses directed to antigen of interest [48]. However, in this work, the objective was to study the IgG responses that were associated with protection to malaria and not the acquisition of the overall IgG response to all polymorphisms of an antigen. Therefore, this study results also show that infant that were producing the highest levels of IgG to MSP3, MSP1 and MSP2 antigens were the ones that were controlling the best the infection. Numerous seroepidemiological studies have previously observed such associations in older individuals [10, 13, 14, 16]. It has been suggested that cytophilic antibodies directed to merozoite antigens are able to limit parasite development through direct inhibition of erythrocyte invasion by merozoites [49–51]. The functional attributes of IgG, using a standardized in vitro growth inhibition assay (GIA), were assessed in samples from 177 infants of this same cohort, but that study failed to show any association between the activity measured by GIA and anti-malarial protection [52]. The analysis of the same GIA data stratified with the groups CAIG/NCIG showed a trend for higher GIA activity in the CAIG group (p = 0.11) suggesting a correlation between quantity and quality of specific IgG response. The effect did not reach the significance probably due to the small number of samples with GIA data (NCIG, n = 64 out of 183 and CAIG, n = 23 out of 53). Interestingly enough, this GIA was performed with purified IgG, meaning with no influence of the complement. First, this suggests that antibody-mediated complement pathway is not the only pathway giving protection and second the effect could be stronger and reach significance in presence of complement as suggested by others [53–55]. Moreover, as shown in some studies, antibodies to *P.* *falciparum* could trigger blood monocytes to control parasite density known as Antibody-Dependent Cellular Inhibition (ADCI) [56, 57] or antibodies to *P.* *falciparum* merozoite surface protein 1p19 could induce antibody-dependent respiratory burst (ADRB) in human neutrophils [58–60].

## Conclusions

The findings presented here showed an association between high levels of cytophilic IgG responses to AMA1 and an increased risk of malaria infection from 6 to 18 months of life. Antibody acquisition to *P.* *falciparum* is highly dependent on parasite exposure and anti-malarial antibodies detected in plasma samples of the study population considered as a whole group represent rather a marker of exposure than a marker of protection. However the group of 53 asymptomatic infants who were able to control asymptomatic malaria infection presented higher antibody levels to AMA1, MSP1, MSP2-FC27, GLURP-R2 and MSP3 than infants from the symptomatic group, underlying the possibility of a very early establishment of naturally acquire immunity against clinical malaria. This last result is encouraging in the context of the development of multitarget *P.* *falciparum* vaccines.

## Data Availability

The datasets used and/or analysed during the current study are available from the corresponding author on reasonable request.

## Abbreviations

IgG1: immunoglobin G 1
IgG3: immunoglobin G 3
ELISA: enzyme linked immuno-sorbent assay
AMA1: apical membrane antigen 1
MSP1: merozoite surface protein 1
MSP2-3D7: merozoite surface protein 2-3D7
MS2_FC27: merozoite surface protein 2-FC27
MS31: merozoite surface protein 3
GLURP R0: glutamate rich protein R0
GLURP R2: glutamate rich protein R2
CAIG: control asymptomatic malaria infection group
NCIG: no control malaria infection group
RDT: rapid diagnostic test
TBS: thick blood smear
IPT: intermittent preventive treatment
ERE: environmental risk of exposure
EDTA: ethylenediaminetetraacetic acid
GPI: glucosylphosphatidylinositol
PBS: phosphate buffered saline
ADAMSEL: Auditable Data Analysis and Management System for ELISA
OD: optical density
CERPAGE: Centre d’Etude et de Recherche sur le Paludisme Associé à la Grossesse et à l’Enfance
IRD: Institut de Recherche pour le Développement
ADCI: antibody-dependent cellular inhibition
ADRB: antibody-dependent respiratory burst
